# Traumatic neuromas of peripheral nerves: Diagnosis, management and future perspectives

**DOI:** 10.3389/fneur.2022.1039529

**Published:** 2023-01-11

**Authors:** Hu Yang, Yanzhao Dong, Zewei Wang, Jingtian Lai, Chenjun Yao, Haiying Zhou, Ahmad Alhaskawi, Sohaib Hasan Abdullah Ezzi, Vishnu Goutham Kota, Mohamed Hasan Abdulla Hasan Abdulla, Hui Lu

**Affiliations:** ^1^Department of Orthopedics, The First Affiliated Hospital, Zhejiang University, Hangzhou, Zhejiang, China; ^2^Zhejiang University School of Medicine, Hangzhou, Zhejiang, China; ^3^Alibaba-Zhejiang University Joint Research Center of Future Digital Healthcare, Zhejiang University, Hangzhou, Zhejiang, China

**Keywords:** traumatic neuroma, peripheral nerve, hand surgery, clinical management, diagnosis

## Abstract

Traumatic neuromas are infrequent in clinical settings but are prevalent following trauma or surgery. A traumatic neuroma is not a true malignancy, rather, it is a hyperplastic, reparative nerve reaction after injury and typically manifests as a nodular mass. The most common clinical manifestations include painful hypersensitivity and the presence of a trigger point that causes neuralgic pain, which could seriously decrease the living standards of patients. While various studies are conducted aiming to improve current diagnosis and management strategies *via* the induction of emerging imaging tools and surgical or conservative treatment. However, researchers and clinicians have yet to reach a consensus regarding traumatic neuromas. In this review, we aim to start with the possible underlying mechanisms of traumatic neuromas, elaborate on the diagnosis, treatment, and prevention schemes, and discuss the current experiment models and advances in research for the future management of traumatic neuromas.

## 1. Introduction

The history of neuromas dates back to 1634 when Ambroise Pare first described the painful neuroma and treated neuroma with massage and oil. In 1811, Odier (1) discovered that the bulbous stump tissue in the proximal ending of a transected nerve could be extremely sensitive. It was not until 1828 that the term “neuroma” was coined by Wood (2), who conducted the first pathological research on such nerve injuries. From then on, the understanding of traumatic neuromas grew steadily, including the introduction of Morton's neuroma by Morton (3), the elucidation of the actual mechanism forming these neuromas by Huber and Lewis (4), and the classification system put forward by Swanson (5), who also defined neuromas as cutaneous and post-traumatic.

A neuroma is not a conventional neoplasm that arises from malignant cells, but a local non-neoplastic proliferation of the injured nerve. Neuromas mostly occur when normal nerve conduction is damaged by injury, inadequate surgical repair, or in some cases, chronic fibro-inflammatory irritation. In this case, the reinnervation process is deterred when nerve reconstruction is interrupted by the interpolation of scar tissue. Depending on the nature of the trauma, traumatic neuromas can be largely categorized into terminal neuroma and neuroma-in-continuity (6). Terminal neuromas are generally observed in limb amputation, lower extremities in particular, where the nerve is completely transected without a distal end, rendering it impossible to reestablish innervation by the injured nerve. On the other hand, neuroma-in-continuity results from a fusiform swelling of the injured site following the trauma and is further divided into two subtypes: (1) total or partial transection of the nerve; (2) repeated blunt trauma to the nerve. The former type of trauma could cause fusiform neuromas, which are commonly seen in the digital nerve and median nerve, while the latter form of trauma causes thickening of the fibrous tissue surrounding the nerve, which is involved in cases of Morton neuroma, Bowler's thumb (7), and lateral femoral cutaneous neuropathy (8). It can be concluded that given the mechanism of traumatic neuromas, it is highly related to traumatic limb injury and occurs mostly in orthopedic patients.

The most common main complaint in patients suffering from peripheral traumatic neuromas remains paresthesia in the innervated area and a painful nodule at the site of injury. Pain, as mentioned before, is among the most common clinical symptoms, including painful hypersensitivity and the presence of a trigger point that causes neuralgic pain. Patients may feel burning, stabbing, raw, gnawing, or sickening sensations. These symptoms could lead to psychological distress and a severe decrease in the quality of life (9). In this review, we aimed to provide a comprehensive understanding of traumatic peripheral neuroma and its related research progress.

## 2. Epidemiology

Traumatic neuromas, as infrequent as it is, are prevalent following trauma or surgery. As previously described, a traumatic neuroma is not a true malignancy. Instead, it is a hyperplastic, reparative nerve reaction after injury and typically manifests as a nodular mass. It is secondary to the abnormal growth of nerves and connective tissue attempting to reinnervate the region following an entire or partial nerve segment due to an accidental or surgical trauma (10, 11). As nerve lesions, traumatic neuromas are characterized as neuroma-in-continuity (NIC) following partial nerve transection or end-bulb neuromas (EBN) following the total disruption (12). Traumatic neuromas exhibit proximal continuity with the parent nerve, similar to the “tail sign” shown in peripheral nerve sheath tumors (PNSTs), indicating a neurogenic origin. On the other hand, EBNs do not present distal continuity with the parent nerve, whereas NICs are continuous both proximally and distally (13).

## 3. Risk factors and pathophysiology

Many studies have found that inhibiting nerve growth factor (NGF) following nerve damage lowers neuroma growth and neuropathic pain in rat models (14). Furthermore, localized deactivation of the brain-derived nerve factor (BDNF) has been found to greatly reduce neuropathic pain and influence the regeneration of sensory fibers. In contrast, excessive concentrations of BDNF promote the growth of neuromas and neuropathic pain (15).

Traumatic damage to a peripheral nerve leads to multidimensional cell proliferation, regeneration failure, and deformed architecture of the nerve. Due to post-traumatic obstruction of axonal flow and subsequent Wallerian degeneration, the nerve segment distant to the site of damage has unique and complicated physiology (16).

Nevertheless, the ability of axons to regenerate and the growing support of Schwann cells (SCs) decrease with time and distance from a trauma (17). On condition that two severed nerve segments are distant respectively, or the proximal end is missing (amputations), axon regeneration occurs in an unstructured manner (18, 19). In some instances, the simultaneous proliferation of wound-healing cells and signaling molecules might result in collagen remodeling and scar formation and eventually forms a neuroma (9). Several studies and case reports have defined traumatic neuroma as having a tangled shape consisting of connective tissue, Schwann cells, and regenerated axons (20–23).

In painful neuromas, inflammatory signaling factors (22, 23) and myofibroblasts (24) have been reported. While the microscopic characteristics of a fully formed neuroma have been thoroughly recorded, little is known about the cellular structure of neuromas in their early stages of development, from nerve damage to initial neuroma formation. Further studies in this field is helpful to generate new and improved therapies that target the earliest stages of neuroma development and prevent the accompanying discomfort (25).

## 4. Diagnosis and clinical evaluation of traumatic neuromas

As a result of injuries or surgical procedures, traumatic neuromas generally present as a firm, oval, slow-growing, palpable nodule with a painful sensation, not larger than 2 cm in diameter. Common symptoms include pain, stiffness, pain hypersensitivity to light tactile stimuli, or neuralgic pain with a trigger point (26). It is vitally important that clinicians pay close attention to the previous medical history of patients with the above symptoms for an accurate initial diagnosis.

Regarding histopathology, Seddon's initial classification of peripheral nerve injuries is based on a three-tiered severity scale: neurapraxia, axonotmesis, and neurotmesis (27). In terms of Electrodiagnosis, axonotmesis and neurotmesis have the same characteristics. The symptoms of axonotmesis are reversible, but those of neurotmesis are irreversible due to disordered axon regeneration. In such instances, complete realignment of the sectioned fascicles and optimum neural tube repair necessitates surgery (28). Following Sudden, the traumatic damage to peripheral nerves was further classified into five classes by Sunderland (29). However, these categories are determined using a presumed prognosis without objective data on the anatomical damage. From this perspective, imaging may be useful for distinguishing between reversible axonotmesis and irreversible neurotmesis.

As trauma majorly affects working-age individuals, delayed management also causes economic and social harm (30, 31). While the classification systems mentioned proved effective in clinical practice, more often than not clinicians are faced with the dilemma that the injured nerve may branch into more regional nerves, even along the length of a nerve the degree of injury may vary. The localization of traumatic neuromas primarily depends on physical examination and medical history, and the application of medical imaging tools. Ultrasound (US) and magnetic resonance imaging (MRI) can be utilized to examine the anatomy and topography of peripheral nerves in order to determine the location, extent, and type of damage (32–37). In examining a limb trauma, it is crucial to examine the probability of nerve structure involvement. A thorough clinical history, physical examination, and electrodiagnostic testing (electromyography—EMG and nerve conduction velocity studies—NCVs) are sufficient to diagnose a nerve injury; nevertheless, a complete qualitative and quantitative assessment of the structural damage is not possible. Determining the kind of anatomical damage and the injury's severity is crucial in deciding whether surgical therapy is necessary since time is crucial for a successful prognosis (38). In addition, a comprehensive morphological diagnosis of a traumatic lesion is crucial in determining optimal care (conservative or surgical treatment) (32, 39). Therefore, the clinical examination strongly recommends morphological imaging using US or MRI (Table 1). The US is a useful utility not only to access the continuity of nerve but also in post-operative follow-up and detection of complications (39). MRI T2-weighted imaging provides high-resolution imaging of peripheral nerve anatomy in combination with fat and flow suppression (34, 41). Many researchers recommend MRI for examining anatomical nerve damage, considering it appropriate for a high-quality assessment (42–45). On the other hand, other researchers believe the two procedures are complementary, favoring the US as an initial examination and MRI for evaluating anatomical regions where US access is difficult or impossible or where US details seem insufficient or do not correspond with clinical suspicion and/or EMG results (33, 36).

**Table 1:** Nerve injury classification following Seddon and Sunderland theories.

**Seddon**	**Sunderland**	**Description**	MRI^*^	Ultrasound^*^
Neuropraxia	I	Conduction block	T2 hyperintensity	The decreased echogenicity of the nerve (hypoechoic)
Axonotmesis	II	Discontinuity of axon with Wallerian degeneration	T2 hyperintensity with increased size Hyperintensity in muscles due to denervation	Decreased echogenicity and increased caliber of the nerve
	III	Scarring of the endoneurium	Endoneurium can-not is delineated with the current MR technique T2 hyperintensity with increased size Hyperintensity in muscles due to denervation	Focal echogenicity decreases with the increased caliber with a change in echotexture of the affected muscles
	IV	A neuroma in continuity with the formation of scar which blocks nerve regeneration	T1 hypointense, T2 hyperintense focal enlargement with loss of fascicular pattern. Hyperintensity in muscles due to denervation	The hypoechoic fusiform lesion is in continuity with the nerve with loss of fascicular architecture and altered echogenicity of denervated muscles
Neurotmesis	V	Rupture of the nerve	End neuroma formation at the proximal end with denervation changes in muscle	Hypoechoic neuroma at the proximal end with local soft tissue edema and denervation changes in muscle
	Mackinnon and Dellon type VI	Mixed injury	Variable findings with nerve heterogeneity and muscle denervation changes	Hypoechoic enlarged with mixed findings of scarring, discontinuity, or neuroma formation

* The data was obtained from Agarwal et al. (40).

**Figure 1:**
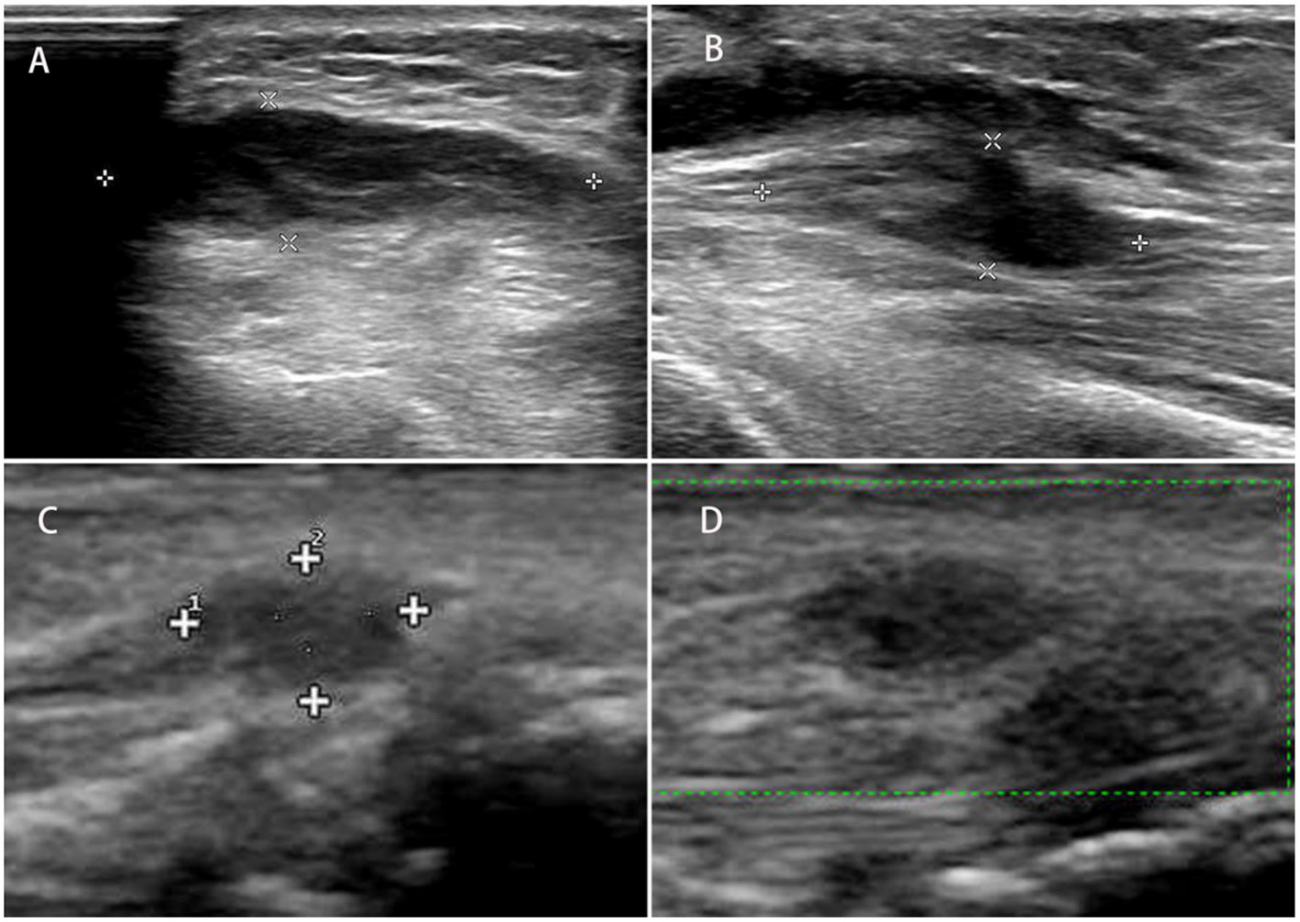
**(A)** Longitudinal and transverse ultrasound images of the left traumatic neuroma of the superficial branch of the radial nerve; **(B)** longitudinal and transverse images of the neuroma; **(C, D)** traumatic neuroma of the superficial branch of the radial nerve of the left wrist.

Currently, few researchers have compared the diagnostic accuracy of the two techniques (36, 46), indicating that both US and MRI can detect the damaged region with a high level of anatomical details and pathological results that correspond with EMG testing. In research by Zaidman et al. (36), it was determined that the US had a greater sensitivity than MRI (93 vs. 67%, respectively), assuming the same level of specificity (86%). In contrast, Aggarwal et al. (46) observed that MRI is more sensitive than US (95 vs. 81%), attributing this to the deployment of a high-field (3 Tesla) MRI scanner; nevertheless, these scanners are not yet accessible for all clinicians. The advantages and limitations of US and MRI are listed in Table 2. The US offers a more affordable alternative, and while well-tolerated by patients, the US shows results in real time. Obtaining dynamic information is also achievable if the patient is requested to undertake certain actions while the physician performs the exam (Figure 1) (60). This dynamic examination provides essential objective data for an overall evaluation of nerve and surrounding tissue damage. On the other hand, high-resolution ultrasound (HRU) is now been identified as a useful tool in the diagnosis of lesions of traumatic neuromas (39, 61–63). HRU can achieve imaging of all main nerves running the limbs including the medial nerve, ulnar nerve, radial nerves, sciatic nerves, common peroneal nerves, and posterior tibial nerves, while still demonstrating the transections, lacerations, hematoma or neuroma formation clearly and accurately (35).

**Table 2:** Advantages and disadvantages of US and MRI in the traumatic damage to peripheral nerves.

**Comparison items**	**Ultrasonography**	**Magnetic resonance imaging**
Spatial resolution	Excellent	Good
Contrast resolution	Good	Excellent
canning planes	Multiple planes (the transducer can be oriented in multiple ways, referring to nerve anatomy)	Multiple planes (2D and 3D acquisitions)
Scan times	Fast	Long
Nerve assessment	Dynamic	Static
Extension of nerve tract examined	Wide, based on need	Limited by the dimensions of the FOV
Denervation changes in muscles	Chronic (atrophy, fatty infiltration)	Early and chronic (edema, atrophy, fatty infiltration)
Deep structures	Limitation	No limitations
Less accessible anatomical areas	Limitation	No limitations
Contraindications	No	Yes
Patient compliance	Good	Poor
Cost	Low	High
Operator dependence	High	Less

The data was obtained and modified after Visalli et al. (47).

Early diagnosis of the location and type of a lesion is crucial for prognosis and treatment since there is a limited clear opportunity for efficient reinnervation and, if fails, the commencement of alternative treatment measures, such as nerve surgery. Nerve recovery begins immediately upon axonal transection when the denervated muscle fibers and skin regions begin producing neurotrophic signals that attract any adjacent surviving axons and induce sprouting of these axons into the denervated tissue (64). The rapid process of reinnervation is known as collateral reinnervation, where the surviving axons in the fascicle innervate the motor units and skin regions of their injured counterparts. In severe nerve damage, when over 75% of the axons in a fascicle is destroyed, the remaining axons in the bundle will be unable to reinnervate every motor unit and skin region, which calls for proximal ingrowth of new axons. The rate of proximal reinnervation is around 1 mm each day, which can be deterred if the length between the nerve lesion and target muscle is too great, as in the case of distal muscles of the lower leg and sciatic nerve injury, since over time there are irreversible alterations in the muscle and neural axon tubes that inhibit further abnormal growth and reinnervation (65). Moreover, injury to the connective tissue constructs of the nerves may result in perineural fibrosis and scarring that can inhibit future axon development. The therapeutic frame for nerve surgery focuses on an effective evaluation of the extent of the injury, which is typically performed clinically and may be enhanced by electromyography (EMG) *via* localizing the exact site of the lesion and the distance between it and the influenced muscle and skin, and identifying morphologic changes of the damaged nerve that indicate transaction or intraneural scarring. By analyzing the regeneration potential, this data will influence surgical decision-making (66).

## 5. Treatment

The process of nerve reconstruction is accompanied by scar formation in the injured site, which eventually results in traumatic neuroma formation (67, 68). Treatment of traumatic neuromas is based on the removal of disturbed nervous tissue or neurolysis to improve the microenvironment surrounding axons and achieving partial or complete remission of painful symptoms (Table 3). Once the neuroma is formed, the priority for clinicians is to relieve symptoms and limit further disease development (54).

**Table 3:** Possible methods during or post-operation for prevention of traumatic neuromas formation.

**Prevention categories**	**Prevention methods**	**Effectiveness**
Surgical skills	Tight ligation, scissors cut, CO_2_ laser, and oblique nerve cutting (48–50)	Good (regular connection between fibers formation)
No nerve gap or free tension injury	Direct nerve repair with or without supporting conduits or gel (51, 52)	Good (depends on surgeon skills)
Nerve gap	Veins autologous, autologous nerve graft (53)	Various (depending on the size of the nerve gap)
Recurrent traumatic neuroma Amputation	Targeted nerve implantation, Terminated nerve stump (49, 54, 55)	Excellent (axons arborize into the intramuscular motor nerve branches)
Nerve support	Nerve conduits, fibrin glue, tacrolimus (FK506) (56, 57)	Various (depending on the severity of the injury)
Others	NGF inhibitor, stem cells, and 3D conduits (58, 59)	Under observation, promising result

### 5.1. Surgical treatment

**Figure 2:**
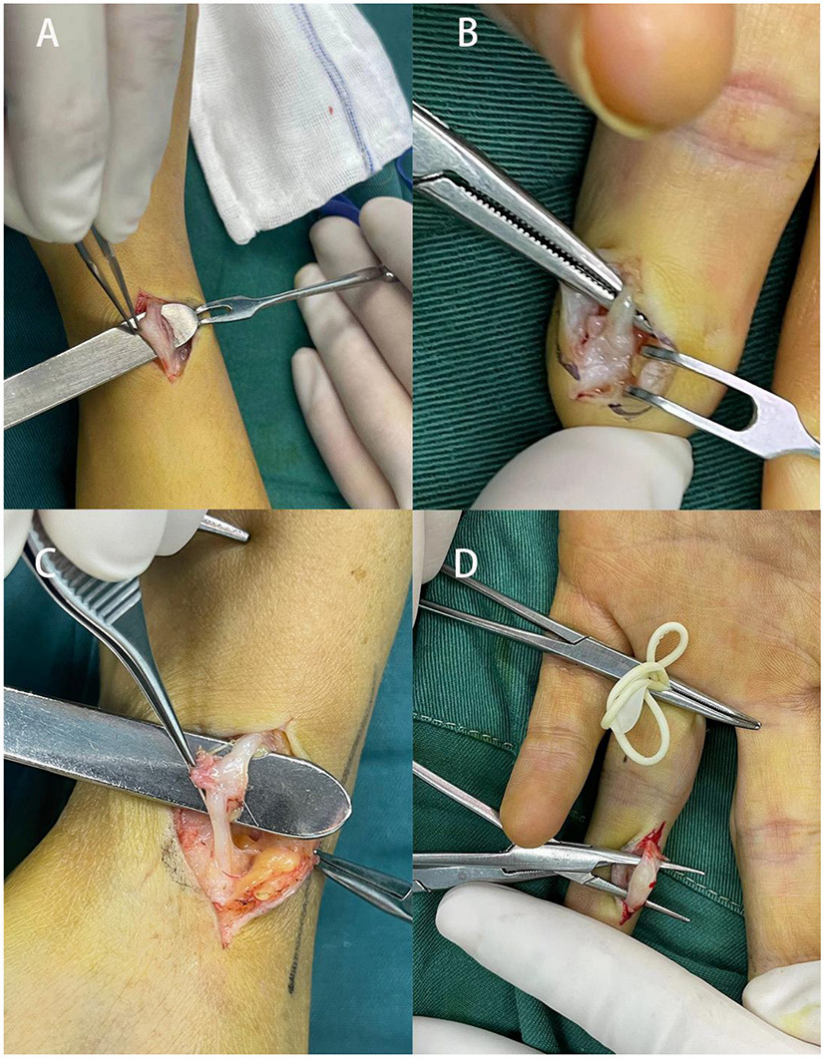
Cases of surgical management in four patients suffering from traumatic neuromas. **(A)** A 32-year-old female patient with a 2-year history of painful mass on the radial side of the right wrist; **(B)** a traumatic neuroma of the middle finger in a 34-year-old female patient as a result of previous finger tope amputation, **(C)** a 24-year-old patient with a traumatic neuroma on the wrist, **(D)** 33-year-old female presented a left index finger traumatic neuroma with local radiation pain for half a month, the patient had a history of left index finger injury by scissors several years ago.

**Figure 3:**
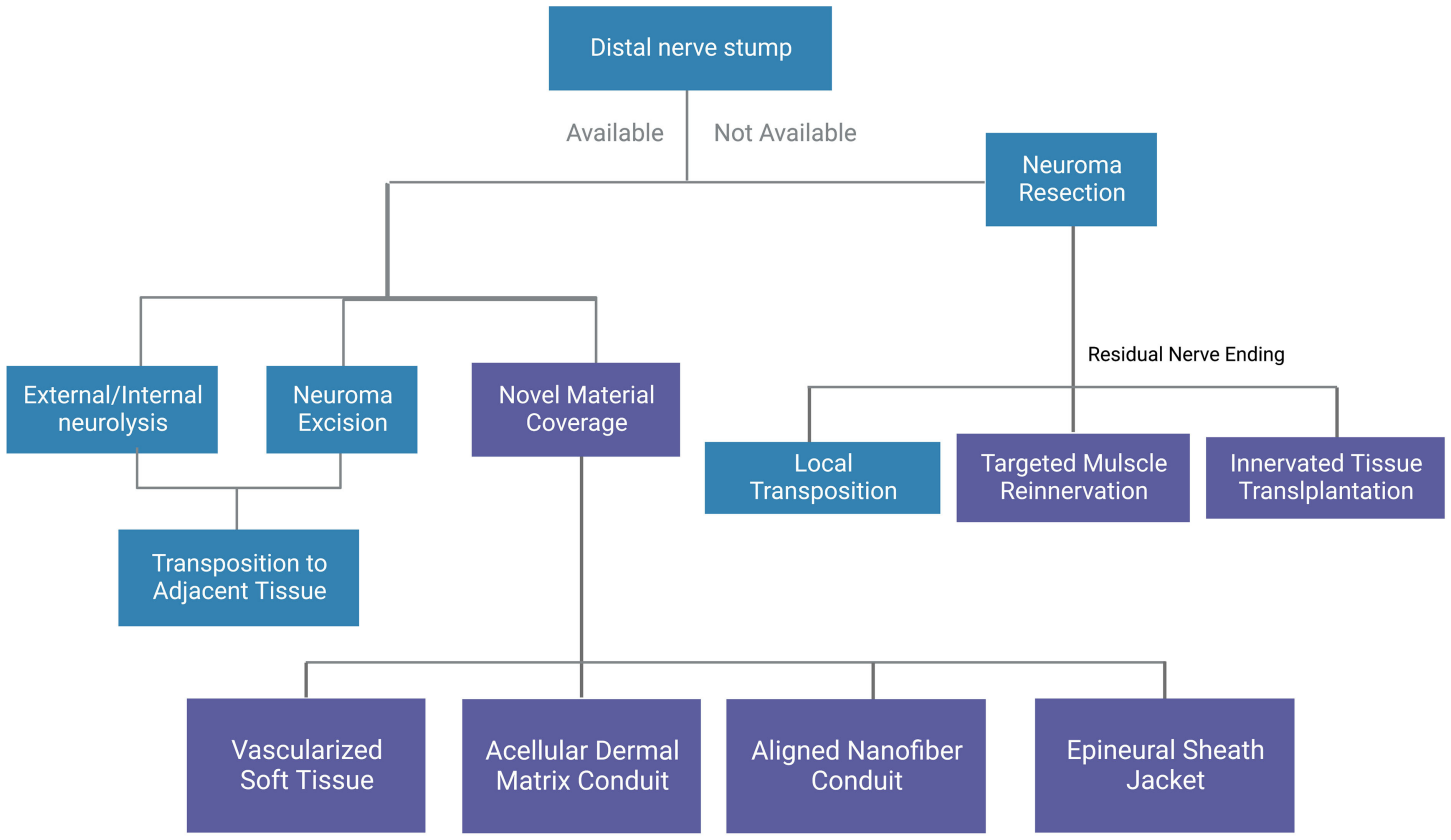
A general summary of surgical methods for traumatic neuromas. Blue boxes indicate conventional methods and purple boxes indicate novel methods.

Surgical treatment is so far the most effective therapeutic method to manage peripheral traumatic neuromas (Figure 2). Various operative techniques have been invented for the management of peripheral traumatic neuroma occurring at different sites (69, 70), including nerve repair, distal nerve ending transposition to muscle, vein, or bone (71, 72), capping of distal nerve-ending with soft tissues or conduits (73–76). In the surgical management of traumatic neuroma, one predominant factor for the selection of operative technique is the continuity of the severed nerve, that is whether there is an adjacent distal nerve stump for future nerve reconstruction and reinnervation. As the described condition is vitally important and greatly affects the selection of operation method, conventional surgical treatment in this section is categorized following this criterion before the proposition of several emerging surgical approaches (Figure 3).

For terminal peripheral neuroma where the distal nerve ending is not available, surgical neurectomy is considered where the target nerve is first identified *via* neurolysis. At this point, palpation of the target nerve should reproduce neuropathic pain in the patient, and resection proximal to the neuroma is performed, followed by transposition of the proximal nerve ending to nearby muscles, bones, and veins, thus reducing connective tissue formation around the nerve post-surgery to obtain a better pain relief effect. This surgical method has been tested out by various researchers and clinicians. In 1984, Mass et al. (77) transposed the hand neuroma into the bone, and 18 of 20 neuromas showed acceptable results. Laborde et al. (72) combined excision of the neuroma with transposition of the proximal palmar cutaneous branch of the median nerve to the pronator quadratus muscle, which freed the nerve from wrist motion and environmental stimulation. Transposition between the superficial and deep flexor muscles is also applicable. Koch et al. (78) studied the resection of the neuroma and transposition of the nerve stump into an adjacent vein, where 22 of 23 patients reported positive long-term results. While the above surgical methods are mainly aimed at nerve trunks such as radial nerve and ulnar nerve, smaller, unknown sensory nerve injuries require a more specific mapping technique. Conventionally, this is achieved by marking the painful area while the patients are awake and applying local anesthetic while incising progressively to identify discrete painful areas (79).

For traumatic peripheral neuroma-in-continuity where the distal ending is available, traditional treatments include external neurolysis, internal neurolysis, excision of the neuroma and transposition of the nerve, etc. External neurolysis or internal neurolysis is used to free the nerve from surrounding scar tissue while maintaining its integrity. Patients with an unsuccessful neurectomy as the primary treatment often suffer from a true neuroma. When the nerve is dispensable, the neuroma-in-continuity can be excised and transposed elsewhere.

Novel treatments targeting neuromas without distal ends mainly include transplantation of innervated tissue to cover the neuroma and targeted muscle reinnervation. In the former type, a muscle flap is always used, Reisman and Dellon (80) adopted a local transfer of the abductor *digiti minimi* muscle flap to treat palmar wrist pain. In the latter type, the cut, distal end of a peripheral nerve is transferred to a nearby muscle target or nerve target, for example, the deep peroneal nerve is transferred to the tibialis anterior muscle and the superficial peroneal nerve is transferred to the peroneus longus muscle (81), interdigital neurorrhaphy for treatment of digital neuromas. In addition to the techniques mentioned above, repeated intervention and higher amputations are rarely advised since it adds to the anxiety and pain of the patient (82). On the other hand, novel treatments for neuroma-in-continuity include the application of vascularized soft tissue or nerve conduits to cover the neuromas. A study by Krishnan et al. (83) showed that vascularized soft tissue coverage of painful peripheral nerve neuromas could be an effective method. Peterson and Adham (84) achieved satisfactory outcomes using an acellular dermal matrix conduit in the treatment of traumatic neuropathic pain at the wrist. Yan et al. (85) found that an aligned nanofiber conduit can significantly facilitate nerve regeneration, inhibit neuroma growth, and reduce traumatic neuropathic pain after neurectomy in a rat sciatic nerve model. Siemionow et al. (86) investigated the efficacy of the epineural sheath jacket as a novel technique for neuroma prevention in the rat sciatic nerve model and gained outcomes comparable to the muscle burying technique. Synthetic conduits may be a promising field in treating traumatic neuroma in the future. In general, traditional techniques combined with novel techniques are more effective in treating traumatic peripheral neuroma. Recently, treatment of traumatic peripheral neuroma has shifted from hiding injured nerves to attempting nerve healing (81). Some literature reviews support this conceptual shift (87, 88). However, more research is required in the future to match suitable operative methods with specific clinical settings.

The numerous techniques that have been attempted suggest that no one technique is completely effective or superior to other techniques, instead, each technique fits the certain situation of nerve injuries. Poppler et al. (89) and Ives et al. (90) compared various surgical treatments for neuroma of the extremities, including excision alone, excision and cap, excision and transposition, excision and repair, neurolysis and coverage, and found no difference in efficacy.

### 5.2. Conservative treatment

Treatment of traumatic peripheral neuroma usually starts with medication. Anti-neuropathic drugs include membrane-stabilizing agents (gabapentin, pregabalin, etc), anticonvulsants [carbamazepine (91), topiramate, phenytoin, lamotrigine, etc], antidepressants (amitriptyline, doxepin, etc), opioid analgesics (pethidine hydrochloride, etc) and muscle relaxants (baclofen, etc). Gabapentin and pregabalin (92) are considered the first-line effective medicine to inhibit central sensitization by affecting the calcium channels and reducing excessive neurotransmitter release. Although anti-neuropathic drugs were used as initial treatment, they often failed to obtain effective results while exposing the patients to side effects.

Other nonoperative management of traumatic peripheral neuroma includes physiotherapy, local injections (anesthetic, steroid, and alcohol), cryotherapy, radiofrequency ablation, shockwave therapy, and electrical stimulation. According to a systematic review from Samaila et al. (93), corticosteroid injections appear to be a safe treatment allowing good results with a very low complication rate. Alcohol injections in a study by Gurdezi et al. (94) showed that although the short-term results are encouraging, alcohol injection does not offer a permanent resolution of symptoms for most patients and can be associated with considerable morbidity. Ultrasound-guided radiofrequency ablation is a novel treatment modality, where a probe is inserted at the site of the neuroma, and the temperature is raised at 85°C for 90 s in an attempt to sever adjacent nerve endings and shows short-term success rates of 80%−85% (95–97).

Friedman et al. (98) performed a retrospective case series review in patients who had undergone sonographically guided cryotherapy for Morton's neuromas, postsurgical and posttraumatic neuromas, and idiopathic neuralgia, where 15 of 20 patients had a positive response to cryotherapy. On the other hand, Friedman et al. (99) did a study of extracorporeal shockwave therapy for an interdigital neuroma in 13 patients and obtained positive therapeutic results.

Continuous high-frequency electrical stimulation can be used to control the hypersensitive state of the injured nerve according to the gate control theory proposed by Wall et al. (100). A study by Stevanato et al. (101) showed that all patients experienced pain relief within a few minutes of treatment (>75 and >95% in most), with long-lasting pain relief with a reduction in mean Numerical Rating Scale of 76.2% after 6 months and of 71.5% after 12 months. No significant adverse events were observed.

## 6. Prevention

Traumatic neuroma patients generally suffer from low life quality caused by function abnormalities, chronic pain syndrome (traumatic neuropathic pain) which may continue for weeks or years, and psychological issues (26, 102, 103). As prevention is better than cure, the first defense line against traumatic neuroma is the prevention of such events. Reconnecting the two ends of the injured nerve after nerve transection is important to obtain a better outcome and decrease the incidence of traumatic neuroma (104).

Several techniques have emerged to prevent traumatic neuroma based on better surgical treatment of peripheral nerve injuries. The incidence of developing traumatic neuroma increases along with the application of electrocoagulation or cryo-neurolysis, compared with other methods such as tight ligation or scissors cut and CO_2_ laser, indicating that applying the right neurectomy method helps to prevent traumatic neuroma (48, 49). Furthermore, some studies have investigated how the direction of nerve cutting can prevent traumatic neuroma formation, and they found that oblique nerve cutting displayed a better outcome compared with perpendicular or transverse cutting. This phenomenon occurred majorly because oblique nerve cutting leaves long and short nerve fibers, where a growth pathway will be formed between them (50). Amputated or untreated peripheral nerve injuries have a higher incidence of traumatic neuroma formation than neurorrhaphy (51). Direct nerve repair, on the other hand, can be divided into epineural repair and grouped fascicular repair and requires a tension-free environment and microscope magnification to obtain better recovery and prevention of traumatic neuromas (52).

NGF was experimentally proved to inhibit NGF decreasing neuroma formation, and minimizing neuropathic pain in traumatic neuroma formation after peripheral nerve injury (14), by applying an antibody against a specific receptor (trkA), or antibody against brain-derived neurotrophic factor (BDNF) which is usually upregulated after nerve injury by NGF (56). Fibrin glue proves its effectiveness in peripheral nerve repair by reducing the inflammatory response and improving axon regeneration. Also, Davis et al. showed that the continuous release of local tacrolimus (FK506) to repair site cloud actively inhibits neuroma formation (57). They used 3D-printing technology to create a polyethylene Y-shape conduit loop, where the nerve will be connected instead of direct connection to autografts. Other studies pointed out that the use of stem cells could improve nerve regeneration and possibly reduce neuroma formation, which could be a potential advanced traumatic neuroma prevention method (58, 59).

## 7. Experimental neuroma model

Experimental research attaches great importance to the treatment and management of traumatic neuromas. Over the years, with an enormous amount of neuroma models proposed in different animals, there is no model that could be of reference, for each model has its own strengths and weaknesses (105).

Rats are the most common species studied in animal models of traumatic neuroma, for the anatomy of rat nerves is well-established and similar to human anatomy (58, 86, 106–109). Besides, the model has a large number of standardized functional tests, making the experimental results easy to evaluate (110, 111). However, one of the major drawbacks of the rat models is that peripheral nerve regeneration is much faster in rodents than in humans, and made worse by the fact that only relative short nerve gaps can be obtained in rodents, making it difficult to compare this *in vivo* model with humans and to further apply treatment into clinical trials. Compared to rats, limiting further use of these mice models.

Rabbits used to be common traumatic neuroma models (112–115). Compared with mice models, rabbits yield longer regeneration time, worse nerve regeneration, and longer gaps which resembles humans more (116). However, the major disadvantage of the rabbit neuroma models is that rabbit nerve anatomy and limb muscle function differ significantly from human beings (117). Although similar to human beings, Primates are rarely used in research because of ethical concerns (118).

According to the research, the sciatic nerve, saphenous nerve (48), sural nerve (119), and tibial nerve (120, 121) can be used for modeling. Among these nerves, the sciatic nerve has gradually become the most common site for the animal model of a traumatic neuroma given the fact that the sciatic nerve is easy to expose and observe, while others are rarely used now (58, 86, 106, 107). After exposure of the sciatic nerve and its trifurcation under the microscope, the nerve is sharply dissected 3–10 mm distal from the trifurcation (108, 113, 114). To prevent spontaneous regeneration of the distal nerve stump, at least 10 mm of the distal nerve stumps should be removed and discarded (106–108). Finally, the presence or absence of a traumatic neuroma was determined by gross observation, ultrasound, hematoxylin-eosin staining, and immunofluorescence (106, 122). After reaching the predetermined time of the experiment or the growth and formation of the neuroma, the animals were sacrificed, and the nerve stump on the severed side and the contralateral normal nerve were harvested for neuroma adhesion evaluation, weight ratios, western blot analysis, and histological analysis.

## 8. Advances in research

In order to cover and cope with the recent advances and findings of traumatic neuromas of peripheral nerves, research has been conducted to improve and decrease the scientific gaps in this issue. Agarwal et al. (40) assessed the role of using imaging tools (high-resolution ultrasonography and MR neurography) and precise anatomical localization in diagnosing peripheral mononeuropathy. With IRB consent, a hospital-based prospective analytical investigation was conducted on 180 peripheral nerves in 131 individuals with symptoms of peripheral mononeuropathy in a context with limited resources. Generally, the proton density fat-saturated MR sequence had the best diagnosis accuracy (93.89%), followed by high-resolution ultrasonography (80%). The proton density fat-saturated sequence showed the maximum sensitivity, whereas the T1 MR sequence had the highest specificity. It was determined that the cumulative diagnostic accuracy of both modalities was 93.33%, with an 80% negative predictive value. Ultrasound and MRI revealed nerve interruption cases, but MRI was more effective at identifying neuromas. With the development of devices with a higher frequency and enhanced MR field strength, imaging of peripheral nerves is more accurate. Nerve imaging permits anatomical delineation and identification of the precise location of involvement. This comparative analysis highlights the significance of imaging in detecting peripheral nerve diseases, with an accuracy of 93.89% for MRI, which might act as the imaging gold standard. High-resolution ultrasonography can serve as a viable screening method since it is faster, more cost-effective, and has an accuracy of 80%, which is equivalent to other diagnostic techniques. The authors concluded that these two imaging techniques are not mutually exclusive. Instead, they complement one another and can be utilized in tandem to diagnose peripheral neuropathies using imaging.

Around 2.8% of hospitalized trauma patients suffer from acute peripheral nerve damage (123). Consequent significant disorder and socioeconomic effects have prompted continued research efforts on this subject (124). If a tension-free direct approximation of the nerve stumps is achievable, the epineural nerve suture is the treatment option. However, if tension-free coaptation is impossible, the current gold standard is autologous nerve transplantation (ANT) (125). Nevertheless, given the restricted accessibility of donor nerves and the morbidity associated with donor sites, new procedures are required to assist in peripheral nerve surgery. Nowadays, it is generally accepted that the material utilized to assist peripheral nerve regeneration should ideally consist of a totally biodegradable matrix that does not negatively impact regeneration during biodegradation (126). Despite significant advances in tissue engineering, no substance or bio-mimicking idea has yet demonstrated improved peripheral nerve regeneration results compared to the ANT, the current gold standard for bridging peripheral nerve deficits (125). In addition to the well-existing substances, chitosan is a relatively potential new substance in peripheral nerve regeneration. Due to its global availability, low cost, complete biodegradability, safe byproducts, and potentially compromising on the regeneration process (127). According to the literature, chitosan was shown to promote axonal regeneration [Kanazawa et al. (128), Stenberg et al. (129)], minimize severe scarring and enhance functional recovery (130), and inhibit further neuroma development peripheral nerve damage (131).

Relying on the processing of chitosan, the degree of acetylation (DOA) might vary, influencing both the molecular weights and solvent properties (132). In addition, the DOA has been found to be a factor that influences the survival, proliferation, and cellular activity of regeneration-supporting cells such as SC (133). However, the accurate adjustments of chitosan matrices remain problematic, as the mechanical rigidity, the biodegradation period, the spatial architecture, and the sterilization process all have the potential to influence the axonal regeneration process and must be considered across the manufacturing system (134). Furthermore, chitooligosaccharide (COS), a byproduct of chitosan, has been discovered to stimulate cell proliferation and inhibit apoptosis in SC, the essential cell for adequate axonal regeneration (135, 136). Furthermore, Wang et al. (137) contributed stimulating effects of COS to an expedited cell cycle leading to enhanced SC proliferation. Additionally, COS boosts the CCL2 production by down-regulating the miR-327 of the SC, resulting in improved migration to the damaged area (138). He et al. (139) examined the anti-apoptotic impacts of carboxymethylated chitosan (CMC) on SC by lowering caspase-3,−9, and Bax activities and enhancing Bcl-2 activities in CMC-treated SC. To protect the SC from oxidative stress, COS led to a decline in malondialdehyde activity and an increase in superoxide dismutase (SOD) activity. Subsequent *in vivo* tests on a rabbit model of axonotmesis revealed that daily intravenous injections of COS for 6 weeks dramatically enhanced peripheral nerve regeneration. Interestingly, the amount of regenerated myelinated nerve fibers, the thickness of the myelin sheath, and the compound muscle action potential (CMAP) as a measure of electrophysiological recovery were considerably greater in COS-treated individuals.

Traumatic neuroma-caused traumatic neuropathic pain has long bothered doctors and patients, scientists widely debate the reasons for traumatic neuropathic pain, and the therapy is difficult. Uncertain is the clinical therapy of painful neuroma. Specialists have developed numerous therapeutic methods in this discipline. However, there is currently no accepted standard treatment (26). Treatment strategies have been explored in animals and people, but pharmacotherapies (antidepressants, antiepileptics) continue to be the core of the care of traumatic neuropathic pain. Nerve stump transpositions into a muscle, vein, or bone are regarded as effective, traditional surgical treatments for persistent conditions, especially using soft tissue (83, 131, 140), and the usage of conduits (24, 131, 141, 142); which reflected an effective potential in traumatic neuropathic pain treatment. In recent decades, novel surgical procedures, including tube-guided nerve capping, electrical stimulation, and adipose autograft, have significantly increased the variety of treatments for traumatic neuropathic pain (143). Balcin et al. (144) hypothesized that nerve transplantation into a vein might limit the growth of painful neuromas. Contrasted the transposition of the nerve stump into a nearby vein or muscle as a surgical therapy for a painful neuroma. According to their pre-operative proportions in the muscle group 3 and 12 months after surgery, translocation into a vein resulted in decreased intensity and evaluating pain levels and enhanced sensory, as measured by the visual analog scale and the McGill pain score. This was connected with greater activity levels and enhanced function. In addition, the transposition of the nerve stump into an adjacent vein is favored over its relocation into a muscle. Myofibroblasts are strongly expressed in neuromas, and it is believed that they contribute to pain by compressing the collagen matrix around the sensitive non-myelinated fibers that proliferate to produce a neuromatous protuberance (83). Krishnan et al. (83) found that covering painful peripheral nerve neuromas with vascularized soft tissue might be an effective but difficult therapeutic strategy. For years, all types of nerve conduits have been used to repair nerve abnormalities (145), and have also been launched to treat painful neuromas (141).

## 9. Conclusion and future directions

Traumatic neuromas have long been a clinical challenge for doctors and researchers. While the standard classification system has been set based on Seddon's and Sunderland's theories, the imaging technology remains in dispute as opinions are divided in selecting the optimal tools among US, MRI, HRUS, EMG, and ect. The diagnosis of traumatic neuromas, with the utility of imaging tools, is based most generally on the previous medical history of nerve injury or operation and symptoms including pain hypersensitivity and the presence of trigger points that causes neuralgic pain. Concerning the management of traumatic neuromas, while surgical procedures are still the most effective treatment method, researchers are eager to develop more non-surgical methods including medication and physical therapy for treatment and prevention of traumatic neuromas. Although clinicians have yet to reach a consensus on a standardized management strategy of traumatic neuromas, it is clear that proper surgical procedures are vital for the prevention of traumatic neuromas. The future perspectives for management of traumatic neuromas, therefore, is most likely the prevention strategy during and post-operation, including an improved surgical approach, or the application of implants with a sustained release of medication that guides proper nerve regeneration.

## Author contributions

HL and HY designed the study. YD, ZW, and JL performed data collection. HZ, AA, and SH analyzed the results. VK, MH, CY, and YD drafted the manuscript. The authors have read and approved the final manuscript.
